# Localization of parathyroid adenomas using ^11^C-methionine pet after prior inconclusive imaging

**DOI:** 10.1007/s00423-017-1549-x

**Published:** 2017-01-14

**Authors:** Milou E Noltes, Annemieke M Coester, Anouk N A van der Horst-Schrivers, Bart Dorgelo, Liesbeth Jansen, Walter Noordzij, Clara Lemstra, Adrienne H Brouwers, Schelto Kruijff

**Affiliations:** 1 Department of Surgery, University of Groningen, University Medical Center Groningen, P.O. Box 30001, Groningen, The Netherlands; 2 Department of Endocrinology, University of Groningen, University Medical Center Groningen, Groningen, The Netherlands; 3 Department of Radiology, University of Groningen, University Medical Center Groningen, Groningen, The Netherlands; 4 Department of Nuclear Medicine and Molecular Imaging, University of Groningen, University Medical Center Groningen, Groningen, The Netherlands

**Keywords:** Minimally invasive parathyroidectomy (MIP), Primary hyperparathyroidism (pHPT), ^11^C-methionine positron emission tomography (^11^C-MET PET)

## Abstract

**Purpose:**

Minimally invasive parathyroidectomy (MIP) is the recommended treatment in primary hyperparathyroidism (pHPT) for which accurate preoperative localization is essential. The current imaging standard consists of cervical ultrasonography (cUS) and MIBI-SPECT/CT. ^11^C-MET PET/CT has a higher resolution than MIBI-SPECT/CT. The aim of this study was to determine the diagnostic performance of ^11^C-MET PET/CT after initial inconclusive or negative localization.

**Methods:**

We performed a retrospective single center cohort study of patients with pHPT undergoing parathyroid surgery after prior negative imaging and later localization by means of ^11^C-MET PET/CT between 2006 and 2014. Preoperative localization by ^11^C-MET PET/CT was compared with later surgical localization, intraoperative quick PTH (IOPTH), duration of surgery, histopathology, and follow-up data. Also, differences in duration of surgery between the groups with and without correct preoperative localization were analyzed.

**Results:**

In 18/28 included patients a positive ^11^C-MET-PET/CT result corresponded to the surgical localized adenoma (64%). In 3/28 patients imaging was false positive and no adenoma was found. In 7/28 patients imaging was false negative at the side of the surgically identified adenoma. Sensitivity of ^11^C-MET PET/CT was 72% (18/25). Duration of surgery of correctly localized patients was significantly shorter compared to falsely negative localized patients (*p* = 0.045).

**Conclusion:**

In an intention to treat ^11^C-MET-PET/CT correctly localized the parathyroid adenoma in 18/28 (64%) patients, after previous negative imaging. A preoperatively correct localized adenoma leads to a more focused surgical approach (MIP) potentially reducing duration of surgery and potentially healthcare costs.

## Introduction

Primary hyperparathyroidism (pHPT) is a common endocrine disorder, with the highest incidence in elderly women [1]. It occurs sporadically, but is also associated with hereditary syndromes such as multiple endocrine neoplasia (MEN) type 1 and 2. pHPT is characterized by hypercalcemia in the presence of high concentrations of PTH, which can lead to abdominal complaints, osteoporosis, kidney stones, muscle weakness, pain, depression and behavioral changes.

Surgery is the only curative and recommended treatment in patients with pHPT usually by means of a minimally invasive parathyroidectomy (MIP). In MIP, surgeons remove the adenoma via a unilateral approach with a minimal invasive incision of 1–2 cm. In 80 to 90% of the pHPT cases, only a single parathyroid adenoma is present, making this surgical strategy usually successful [2]. However, to be able to perform a unilateral MIP, accurate preoperative imaging is essential.

Worldwide, the current primary preoperative localization imaging standard consists of cervical ultrasonography (cUS) combined with ^99m^Tc-methoxyisobutylisonitrile single-photon emission computed tomography/computed tomography (MIBI-SPECT/CT) [3, 4]. Planar MIBI scintigraphy has the lowest sensitivity, around 70% [5, 6], performing better when combined with SPECT/CT [5].

However, although better, even MIBI-SPECT/CT alone is not optimal with a sensitivity of 85% [5, 7–13]. But when the MIBI-SPECT/CT is combined with cUS (sensitivity from 22 to 82%) [2, 9–11] a sensitivity of 80–90% can be achieved [14–16]. This means that using these two modalities still in 10–20% of the cases, the surgeon will not be able to schedule the patient for a focused MIP operation.

^11^C-methionine positron emission tomography/CT (^11^C-MET PET/CT) is a nuclear imaging technique that can be used as a next step for imaging after prior negative localization. C-methionine accumulates in the parathyroid adenoma and is involved in the synthesis of the precursor of PTH [17–19]. It improves the detection performance of parathyroid tissue due to a better spatial resolution of PET/CT [20]. When ^11^C-MET PET/CT became available in 2006 at our institute, we used this nuclear imaging modality as a step up approach after inconclusive imaging.

The aim of this study was to determine the diagnostic performance of ^11^C-MET PET/CT after prior negative localization in patients with pHPT.

## Material and methods

This is a retrospective single center cohort study of patients with biochemically proven pHPT who underwent parathyroid surgery after localization by means of a ^11^C-MET PET/CT in a teaching and tertiary referral hospital.

## Patients

The medical charts of all patients who underwent ^11^C-MET PET/CT between January 2006 and December 2014 were reviewed. To be eligible for inclusion, patients had to be older than 18 years and had to have a biochemically confirmed pHPT, for which parathyroid surgery was planned. A MIBI-SPECT/CT and/or cUS had to have been performed, however with negative or inconclusive result, after which patients underwent a ^11^C-MET PET/CT. Patients were excluded if they were known to have a germline mutation predisposing for multiple gland disease or if an alternative diagnosis (e.g., parathyroid carcinoma) was known before surgery.

The medical charts were reviewed to determine the outcome of the imaging tests (negative = no localization stated in the original report, inconclusive = an original report describing a presumed adenoma with doubt), or positive = original reports describing the location of the presumed adenoma without any doubt). Also, data on gender, age, preoperative PTH, corrected calcium, intraoperative quick PTH (IOPTH), previous parathyroid surgery, the length of surgery and pathology outcome were collected. Corrected calcium was calculated using the following formula: Ca + ((40 – Alb) × 0.02). “Ca” is the serum calcium (mmol/l) and “Alb” is the serum albumin (g/l). The diagnosis pHPT was made by experienced endocrinologists from our center and all the patients were discussed in a multidisciplinary endocrine board.

Data obtained from patient records were anonymously stored using study-specific patient codes in a password protected database. The local ethical board evaluated the study and according to Dutch law, no additional review board approval was required.

## cUS

cUS was performed in a number of different hospitals on various ultrasound systems. All patients who underwent cUS were examined in a supine position with a hyperextended neck using a high-frequency linear transducer, as is common practice. The neck was always examined from the level above the thyroid to the clavicle caudally. Findings suggestive for parathyroid adenomas were documented in two planes with special regard to size and anatomic correlation to adjacent structures.

## MIBI-SPECT/CT

Between 2006 and 2014, parathyroid imaging was performed with various protocols due to changes in gamma cameras and radiotracers. Also, some procedures were performed in other hospitals according to slightly different imaging protocols. However, all protocols adhered to the international guidelines [21]. At the University Medical Center Groningen (UMCG) until 2010, images were performed using a Multispect 2 gamma camera (Siemens), on which only SPECT images could be made. Afterwards, patients were scanned on a Symbia T16 gamma camera with CT (Siemens), resulting in SPECT/CT images. Furthermore, always ^99m^Technetium (^99m^Tc)-sestamibi (MIBI) was used for preoperative localization as “dual phase” technique. This technique was always combined with a “dual tracer” subtraction technique for thyroid only visualization, although a switch in tracer was made in 2012, and ^123^I was replaced by ^99m^Tc-pertechnetate. Thus, currently the MIBI-SPECT/CT imaging protocol includes early and late planar MIBI images combined with ^99m^Tc-pertechnetate planar subtraction images, and late MIBI SPECT/CT 3D images.

## ^11^C-Met Pet/CT

In the current study, two PET cameras were used. Patients were either scanned on a Ecat EXACT HR + PET only system or a PET/CT (Biograph mCT, 64 slice CT) camera (in use since October 2009) (both Siemens) and had to fast for 6 h while drinking 1 l of water prior to the PET procedure. PET images were taken 20 min after injection of 7 MBq/kg ^11^C–methionine which was produced on site as described by Phan et al. [22]. The head and neck area was scanned in two bed positions (2D imaging, 7 min per bed position and 2 min transmission scan) using the HR + camera, while it was scanned in three bed positions using the mCT camera (3 min per bed position, prior low dose CT for attenuation correction; 100 kV, 30 Quel Ref mAs, 1.5 pitch). PET images were iteratively reconstructed using four iterations, 16 subsets with a 5 mm Gausian filter for HR+, and using three iterations, 21 subsets with 4 mm Gausian filter for mCT. Low dose CT images were reconstructed with 2 mm slice thickness.

### Surgery

All the surgical procedures were performed by the same three experienced surgeons.

Generally, the focused approach utilized was a mini-incision procedure involving a 2- to 3-cm keyhole incision either laterally at the medial edge of the sternocleidomastoid muscle or centrally, depending on the surgeon’s preference. The aim of the operation was to identify and remove a parathyroid adenoma concordant with the MET-PET imaging, and the ipsilateral gland was not routinely examined.

Failure to locate an adenoma, or an incidental finding of two enlarged ipsilateral glands, would prompt conversion to bilateral 4-gland exploration. IOPTH was measured at T0 (incision), T1 (after removal of adenoma), T2 (+5 min), T3(+5 min) and T4 (+5 min). A successful procedure (final outcome) was defined as a decrease of the IOPTH of at least 65% in the surgical report and the finding of parathyroid tissue in the pathology report. Data on how the surgical procedure was performed, were reviewed by an independent endocrine surgeon unaware of the outcome. Furthermore, final localization of the adenoma during surgery was based on the anatomic description in the surgical report. Follow-up data at 6 months postoperatively were collected (for overall cure rate) to determine if patients were cured or still experienced pHPT with symptoms.

Preoperative adenoma localization by ^11^C–MET-PET/CT was defined as true positive, true negative, false positive, and false negative, dependent on the final outcome. The final outcome was based on the surgical and pathology report. Suspected adenomas localized to the correct side (left or right) on the basis of surgical and pathologic findings were scored as true positive. Suspected adenomas localized to the incorrect side were scored as false positive. Sensitivity of ^11^C–MET-PET/CT was calculated as the number of true positive scans divided by the true positive and false negative scans, at a patient level. The duration of surgery in minutes (min) was determined per group, depending on the ^11^C-MET PET/(CT) results.

## Statistics

Data were analyzed using descriptive statistics on a patient based level. Mean (± SD) or median with range were calculated when appropriate. Differences between duration in surgery in different groups were calculated using a Mann-Whitney *U* test. Categorical variables were expressed in proportions. SPSS version 22 statistical software was used. A *p* value of <0.05 was considered significant.

## Results

### Patients

In total, 65 patients underwent a parathyroid ^11^C-MET PET/CT between January 2006 and December 2014. Of these 65 patients, 28 patients were included in this analysis. Patients were not eligible for inclusion because of age < 18 years (*n* = 1), tertiary HPT (*n* = 2), preoperative diagnosis of a parathyroid carcinoma (*n* = 6), patients with a known MEN 2a syndrome (*n* = 3), 1 patient with a lithium based HPT, and 1 patient with familial hypocalciuric hypercalcemia (FHH). An additional 15 patients were excluded because they were not operated. These were patients who did not meet criteria for surgery according to the guidelines (*n* = 9) [3], or patients in whom surgery was deferred due to health risks or the wish of the patient (*n* = 6). Eight patients were referred to our hospital solely for the ^11^C-MET PET/CT and treatment and other clinical data were not available. Of the 15 patients not referred for surgery, ^11^C-MET PET/CT was negative in 11 patients.

Thus, 28 patients were included in this study of which 22 were female. Median age was 68 years (23–84). Patients’ baseline characteristics are depicted in Table 1, including indications for surgery according to the guidelines [3].

**Table 1:** Patients’ baseline characteristics

Number	Gender	Age	Preoperative PTH (pmol/l)	Corrected calcium (mmol/l)	ASA classification	Previous parathyroid surgery (Yes/No)	Indication for surgery
1	F	41	13.0	2.62	2	Y	RBD, Age, Symp
2	F	67	31	2.79	2	Y	Ca, RBD, Symp
3	M	71	179	3.94	3	N	Ca, GFR, Symp
4	F	66	18.0	3.06	1	Y	Ca, RBD, Symp
5	F	84	121.0	3.30	2	Y	Ca, GFR, Symp
6	M	68	12.0	2.63	2	N	RBD, Symp
7	F	73	21.0	2.96	2	N	Ca, RBD
8	F	46	19.0	2.58^a^	1	N	Age, RS, Symp
9	M	62	27.0	2.98	2	Y	Ca
10	F	51	12.0	2.75	3	N	Ca
11	F	79	34.0	3.23	2	Y	Ca, Symp
12	M	71	8.6	2.72	3	N	RS, Symp, Ca
13	F	68	48.0	2.64	2	N	Symp
14	F	72	22.0	2.83	1	N	RBD, Symp, Ca
15	F	65	44.0	3.11	2	N	Ca, RS, Symp
16	F	60	50.0	2.94	2	Y	Ca, Symp
17	F	23	9.8	2.63	2	N	Age, RS, Symp
18	F	53	16.0	2.72	3	N	Ca, Symp
19	M	72	29.0	2.98	2	N	RBD, Ca, Symp
20	F	76	24.0	2.69	2	Y	RBD, Symp
21	F	81	16.0	2.72	3	N	Ca, Symp
22	F	64	10.0	2.66	2	Y	Ca, Symp
23	M	52	7.2	2.63	1	N	RS, Symp
24	F	33	21.0	2.71	2	N	Ca, RBD, Symp
25	F	71	16.0	2.93	2	Y	Ca, RS, RBD, Symp
26	F	72	62.0	3.66	1	N	GFR, Symp, Ca
27	F	81	19.0	2.74	2	N	GFR, Ca, Symp
28	F	62	17.0	2.82	1	N	Ca, Symp

*F* female, *M* male, *Y* yes, *N* No, *RBD* reduction bone density (women/men >50 with a *T* score of ≤2.5 at the lumbar spine, femoral neck, total hip, or 33% radius. Women/men <50 with a *Z* score ≤2.5), *Age* age younger than 50 years, *Symp* symptoms, *Ca* elevated calcium (>0.25 mmol/l above the upper limits of normal), *GFR* GFR <60 ml/min 1.73 m2, *RS* renal stones

^a^This patient showed an elevated calcium excretion in a different hospital. Also, we assume that serum calcium was most elevated at time of diagnosis. However, this has not been well documented in the patient record at the UMCG

Table 2 shows the results of the preoperative localization techniques and results, the intraoperative findings, and findings on pathology and final diagnosis. A cUS was performed in 16 of the 28 patients, of which in 10 patients the cUS was performed outside our center. Of these cUS, in 3 patients the results are unknown. In 7 of the remaining patients the cUS did not show an adenoma (negative), in 6 patients results were inconclusive.

**Table 2:** Patients’ results with preoperative localization, intraoperative, and pathological findings, diagnosis, and follow-up

Patient number	Clinical diagnosis	cUS	MIBI-SPECT/(CT)	^11^C-MET PET/(CT)	Intraoperative findings	IOPTH	Pathology	Diagnosis	Follow-up
1	pHPT	N.A.	Negative	Left	Left	Decreased	Adenoma	pHPT	Cured
2	pHPT	Negative	Negative	Left	Left	Decreased	Adenoma	pHPT	Cured
3	pHPT	Incon bilateral	Incon right	Right	Right	Decreased	Adenoma	pHPT	Cured
4	pHPT	Incon right	Negative	Right	Right	Decreased	Adenoma	pHPT	Cured
5	pHPT	N.A.	Incon left	Left	Left	Decreased	Adenoma	pHPT	Cured
6	pHPT	Negative	Negative	Negative	Left	Decreased	Adenoma	pHPT	Cured
7	pHPT	Negative	Negative	Negative	Right	Decreased	Adenoma	pHPT	Cured
8	pHPT	Negative	Negative	Right	Right	Decreased	Adenoma	pHPT	Cured
9	pHPT	N.A.	Incon right	Right	Right	Decreased	Adenoma	pHPT	Cured
10	pHPT	N.A.	Negative	Right	Right	Decreased	Adenoma	pHPT	Cured
11	pHPT	N.A.	Negative	Right	Right	Decreased	Adenoma	pHPT	Cured
12	pHPT	N.A.	Negative	Left	Left	Decreased	Adenoma	pHPT	Cured
13	pHPT	N.A.	Incon left	Left	Left	Decreased	Adenoma	pHPT	Cured
14	pHPT	Negative	Negative	Negative	Right	Decreased	Adenoma	pHPT	Cured
15	pHPT	Incon right	Negative	Right	Right	N.A.	Adenoma	pHPT	Cured
16	pHPT	N.A.	Incon right	Bilateral	Negative	Not decreased	Not aplicable	Persistent hyper-parathyroidism	Not cured
17	pHPT	N.A.	Negative	Left	Right	Decreased	Adenoma	pHPT	Cured
18	pHPT	Negative	Negative	Negative	Right	Decreased	Adenoma	pHPT	Cured
19	pHPT	Incon right	Negative	Right	Right	Decreased	Carcinoma	Parathyroid carcinoma	Cured
20	pHPT	N.A.	Negative	Right	Negative	Not decreased	Not aplicable	Persistent hyper-parathyroidism	Not cured
21	pHPT	Negative	Incon right	Right	Right	Decreased	Adenoma	pHPT	Cured
22	pHPT	Incon right	Incon right	Right	Negative	Not decreased	Papillary thyroid carcinoma	Persistent hyper-parathyroidism	Not cured
23	pHPT	N.A.	Incon left	Right	Right	Decreased	Adenoma	pHPT	Cured
24	pHPT	N.A.	Negative	Negative	Right	Decreased	Adenoma	pHPT	Cured
25	pHPT	Incon bilateral	Incon left	Right	Right	Decreased	Adenoma	pHPT	Cured
26	pHPT	N.A.	Negative	Bilateral	Left	N.A.	Adenoma	pHPT	Cured
27	pHPT	N.A.	Negative	Left	Left	Decreased	Adenoma	pHPT	N.A.
28	pHPT	N.A.	Left^a^	Negative	Left	Decreased	Adenoma	pHPT	Cured

*N.A*. not available, *Incon* inconclusive

^a^MIBI-SPECT/CT was negative at first, but after performance of ^11^C–MET PET/CT the MIBI-SPECT/CT was revised as positive on the left side

MIBI-SPECT/CT was performed in all 28 patients. Twelve patients underwent MIBI-SPECT/CT according to the old protocol (late ^123^I images subtracted from the early ^99m^ Tc-sestamibi), while 8 patients underwent MIBI-SPECT/CT according to the current protocol (^99m^Tc-pertechnetate images subtracted from the early ^99m^Tc-sestamibi images), and in the other 8 patients, MIBI-SPECT/CT was performed outside our center. MIBI-SPECT/CT was negative in 18 patients and inconclusive in 9 patients. In 1 patient, MIBI-SPECT/CT was negative at first, but after revision at the time of ^11^C-MET PET/CT, the MIBI-SPECT/CT images were scored as positive on the left side.

### ^11^C-MET PET/CT and surgical results

**Fig. 1:**
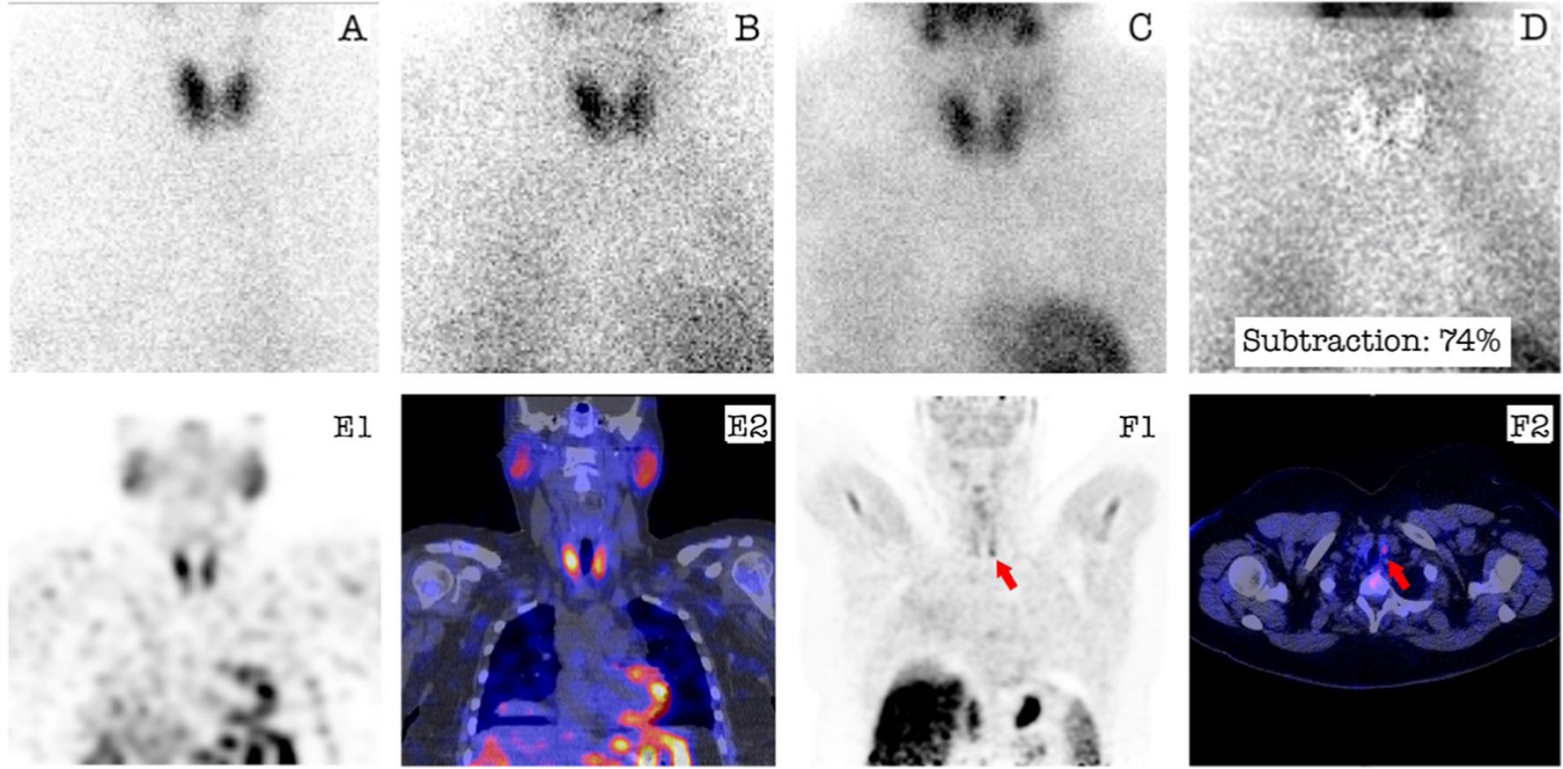
Patient example of a negative MIBI-SPECT/CT and a positive ^11^C-MET PET/CT. Planar anterior image of the neck with ^99m^Tc-pertechnetate (**a**), early ^99m^Tc-MIBI (**b**), and late ^99m^Tc-MIBI (**c**). Both planar subtraction image (early ^99m^Tc-MIBI minus ^99m^Tc-pertechnetate image (**d)**) and ^99m^Tc-MIBI SPECT-CT (E1-SPECT image only and E2 fused SPECT/CT image) do not show a clear focus suspect for adenoma. The ^11^C-MET PET/CT showed a small lesion located caudally from the left thyroid gland, suspicious for parathyroid adenoma (*red arrow* F1-PET image only and F2-fused PET/CT image)

Table 3 shows the results of the final outcome compared with the preoperative localization by ^11^C-MET PET/CT. The HR + camera (PET only) for ^11^C-MET PET was used in 11 patients. The mCT PET/CT camera was used in 17 patients. ^11^C-MET PET/CT was positive in 21 patients and negative in 7 patients. Figure 1 shows an example of a patient (number 1) with a negative MIBI-SPECT/CT and a positive ^11^C-MET PET/CT.

**Table 3:** Final outcome of surgery and pathology combined compared with results of ^11^C-MET PET/CT

	Surgery + Pathology	
^11^C-MET PET/CT	Positive	Negative
Positive	18	3
Negative	7	0

^11^C-MET PET/CT was true positive in 18 of the 28 patients (64%) (6 on the left side, 11 right side). In 1 patient, ^11^C-MET PET/CT was positive bilaterally, but during surgery the adenoma was localized on the left side.

In 3 of the 28 patients, ^11^C-MET PET/CT was positive, while the surgeon could not locate the adenoma during surgery. In 2 of these 3 patients, imaging was positive on the right side and in 1 patient imaging was positive bilaterally. These 3 patients were all diagnosed with persistent mild hyperparathyroidism during follow-up for which no medical treatment (specifically cinacalcet), was deemed necessary. In one patient, accidentally a papillary thyroid carcinoma was found in the pathology specimen. This may have caused the false positive ^11^C-MET PET/CT [22]. Even after completion of thyroidectomy, the (mild) hyperparathyroidism still persisted and a parathyroid adenoma was never found. [22].

In the remaining 7 patients, ^11^C-MET PET/CT was false-negative at the side where the surgeon located the adenoma. In these patients, the surgeon did not perform a focused approach, but a unilateral or bilateral neck exploration. In 6/7 patients imaging was negative, but during surgery the adenoma was found on one side (two on the left, four right). In 1/7 patients imaging was positive on the left side, but during surgery, the adenoma was found on the contralateral side.

In the 25 patients with positive surgery, IOPTH showed a decrease of at least 65% in 23 patients. In two patients the IOPTH was not performed, because these patients were operated outside our center, while pre-operative imaging and follow-up was performed in our center.

The sensitivity of ^11^C-MET PET/CT calculated at a patient level was 72% (18/25).

Of the 18 true positive findings, surgeons explored unilaterally in 12 patients, bilaterally in 4 patients and in 2 patients data is unknown. Of the 7 false negative findings, surgeons explored unilaterally in 2 patients and bilaterally in 5 patients. In the 3 false positive findings, surgeons explored unilaterally in 1 patient and bilaterally in 2 patients.

In 25 (18 true positive +7 false negative) patients, the surgeon found a suspected adenoma. Histopathology confirmed a parathyroid adenoma in 24 patients and in 1 patient histopathology confirmed an accidentally found parathyroid carcinoma (Table 2).

Of the 25 patients with positive surgery, 25 were cured and did not experience symptoms during follow-up resulting in an overall cure rate of 86% (24/28) as one patient was lost during follow-up (Table 2).

### Duration of surgery

A significant difference was present in the duration of surgery between patients with a true positive ^11^C-MET PET/CT compared to patients with a false negative ^11^C-MET PET/CT. True positive patients spend a median time of 194 min (128–281) in the operation room, while false negative patients spend a median time of 237 min (190–269) in the operation room (*p* = 0.045).

## Discussion

This retrospective single center cohort study evaluates the diagnostic performance of ^11^C-MET PET/CT after prior non-conclusive localization via MIBI-SPECT/CT and/or cUS in patients operated for biochemically confirmed pHPT. In this challenging clinical setting, ^11^C-MET PET/CT was able to adequately localize the adenoma correctly in nearly two-third of the patients. Thus, in these patients the surgeon was subsequently able to perform a successful focused approach.

We found that ^11^C-MET PET/CT in this series localized the adenoma in 64% at the correct side of the neck. In 3 patients, the ^11^C-MET PET/CT was false positive, and the surgeon could not locate an adenoma. Reasons for false positivity might be benign and malignant thyroid lesions, as was the case in one of our patients [22].

Although few studies describe the diagnostic performance of ^11^C-MET PET/CT, our sensitivity of 72% is comparable with earlier studies. Beggs et al. who included 51 patients, found a sensitivity of 83% [23]. In Beggs et al., also patients in whom other imaging techniques such as MIBI scanning, CT, or ultrasound had earlier failed to localize the adenoma were selected. In a recent smaller study with 18 patients, Braeuning et al. found a sensitivity per patient of 91.7% and a sensitivity per lesion of 73.3% after a prior negative ^99m^Tc-MIBI-SPECT/CT [24]. Another smaller study by Chun et al. who included 16 patients found a sensitivity of 91.7%, but did not select patients with prior negative imaging [20]. Traub-Weidinger et al. included 15 patients with negative ^99m^Tc-MIBI SPECT/CT and earlier neck surgery because of pHPT and/or thyroid disorder and found a sensitivity of 40% [25]. With a sensitivity of 40% for ^11^C-MET PET/CT, these results differ from other studies. The varying surgical history of the patients in this series may be an explanation [25]. Martinez-Rodriguez et al. included 14 patients in a prospective study and showed a sensitivity of 76.9%, but only 2 patients had previous neck surgery [26]. Additionally, no patients with prior negative imaging were selected. So, with 28 included patients (10 with previous neck surgery) this is one of the largest studies reflecting the performance of ^11^C-MET PET/CT in patients with pHPT after prior negative imaging.

We realize that our retrospective study has limitations. In this study, only patients were included who had undergone ^11^C-MET PET/CT and were subsequently surgically treated. Correspondingly, of the 15 patients not referred for surgery, ^11^C-MET PET/CT was negative in 11 patients. This referral bias may have led to overestimation of the sensitivity of the ^11^C-MET PET/CT. However, our study group also included patients with negative results of ^11^C-MET PET/CT that underwent surgery. Also, performance of MIBI-SPECT/CT highly depends on the protocol used. If all patients would have received a dual phase MIBI-SPECT/CT, more adenomas could have been localized and in the remaining patients, adenomas might have been harder to localize. This may also have led to some overestimation of the performance of ^11^C-MET PET/CT.

In this study, the results of surgery were seen as the gold standard, which could be a drawback of the current analysis. Although a surgical neck exploration is technically protocolled, heterogeneity in surgical techniques remains inevitable. Also, in this study imaging procedures changed slightly during the years, which might have influenced our results.

The strong point of our study is that it describes and reflects the strategy of preoperative ^11^C-MET PET/CT after earlier negative imaging. The success of a focused approach strongly depends on how well the adenoma is localized by imaging preoperatively.

In this series duration of surgery is relatively long, since anesthesiology and waiting time for IOPTH was included and the data were gathered in a tertiary referral hospital which adds to more extended surgery times because of a patient selection for more difficult cases. Furthermore, we conduct a training program for residents and therefore also provide surgical training during operations which required time because of learning curve. Most importantly however, if ^11^C-MET PET/CT correctly located the adenoma, the duration of surgery was significantly shorter.

In general, preoperative localization saves operation time because of a double effect. Firstly, operating via keyhole surgery is a shorter and more effective procedure compared to an open bilateral exploration and secondly the surgeon knows where the adenoma should be located. Furthermore, if the surgeon finds the adenoma where it was expected to be, often less perioperative adjuncts such as IOPTH are needed, also shortening the procedure. Finally, when the patient is informed about the small risk of having a 5% chance of a second adenoma, a second contralateral procedure in the future will still be in a surgical virgin territory and therefore is an acceptable calculated risk. We speculate that eventually in the long run, this might lead to a decrease in healthcare costs. More research on this topic is mandatory.

Globally, cUS and MIBI-SPECT/CT are used first in line to localize parathyroid adenomas being relatively cheap and widely available imaging techniques [3, 4]. Replacing cUS and MIBI-SPECT/CT with ^11^C-MET PET/CT is not a realistic option. ^11^C-MET PET/CT has a more complicated radiochemistry tracer production, includes the need for a cyclotron on site, and has significantly higher costs. When available, it is therefore more realistic to use ^11^C-MET PET/CT as a step up approach only after earlier negative localization by MIBI-SPECT/CT and/or cUS.

Other various imaging protocols in the detection of a parathyroid adenoma are also feasible. For example, four-dimensional computed tomography (4DCT) is another reported strategy as a next step imaging procedure, because of its high sensitivity (88%) [27, 28]. Choline PET, known from its use in prostate cancer diagnostics, can either be performed with ^11^C–choline or ^18^F–choline and may be a good imaging alternative for ^11^C-MET PET/CT. First results in literature look promising [9, 29, 30], however, results comparing the two tracers directly are not available. Also, in this area more research is warranted. Furthermore, a cost-effectiveness analysis on the various anatomical and nuclear imaging techniques and the order in which to use them in the setting of pHPT could also be performed.

## Conclusion

In conclusion, this retrospective analysis shows ^11^C-METPET/CT to be a sensitive method for the localization of parathyroid adenomas in patients with clinically suspected pHPT (sensitivity of 72%). ^11^C-MET PET/CTcorrectly detects parathyroid adenomas in 64% of operated patients after prior negative MIBI-SPECT/CT and/or cUS. Since ^11^C-MET PET/CT is able to additionally localize adenomas, resulting in more patients who can be operated via MIP, the duration of surgery and thus healthcare costs potentially decrease.
